# Supplementary material for: Chimeric Stimuli-Responsive Liposomes as Nanocarriers for the Delivery of the Anti-Glioma Agent TRAM-34
Source: Int J Mol Sci. 2021 Jun 10;22(12):6271. doi: 10.3390/ijms22126271 (PMC8230631; doi:10.3390/ijms22126271)
Supplement: Supplementary file 1 [file ijms-22-06271-s001.zip › ijms-1239447 supplemenary.pdf]

Supporting Information

Chimeric Stimuli-Responsive Liposomes as Nanocarriers for the Delivery of the Anti-Glioma Agent TRAM-34

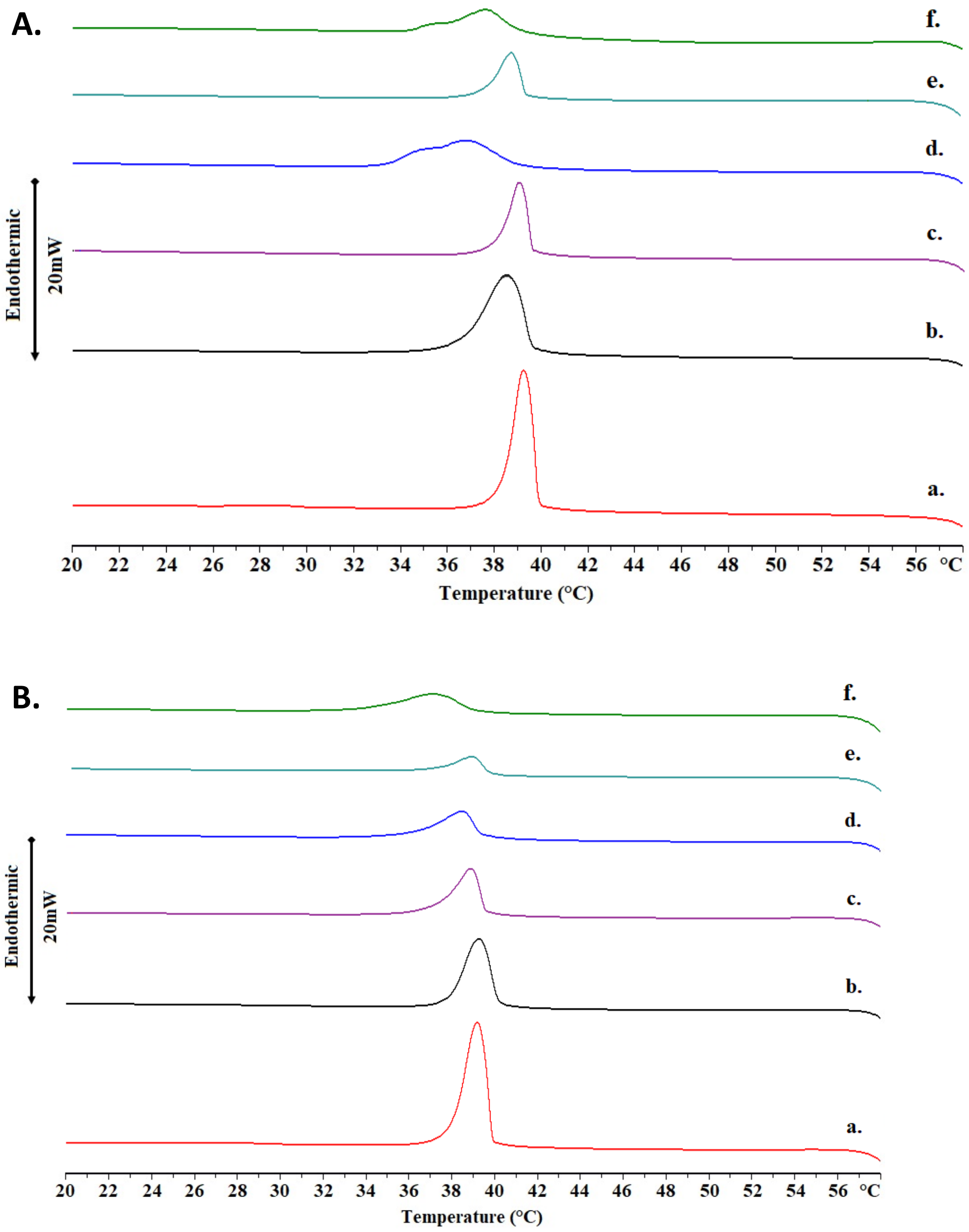

Figure 1. DSC cooling curves in A. PBS (pH = 7.4) and B. citrate buffer (pH = 4.5) of a. DPPC, b. DPPC:Tram-34, c. DPPC:PDMAEMA-b-PLMA 1, d. DPPC:PDMAEMA-b-PLMA 1:Tram-34, e. DPPC:PDMAEMA-b-PLMA 2 and f. DPPC:PDMAEMA-b-PLMA 2:Tram-34.

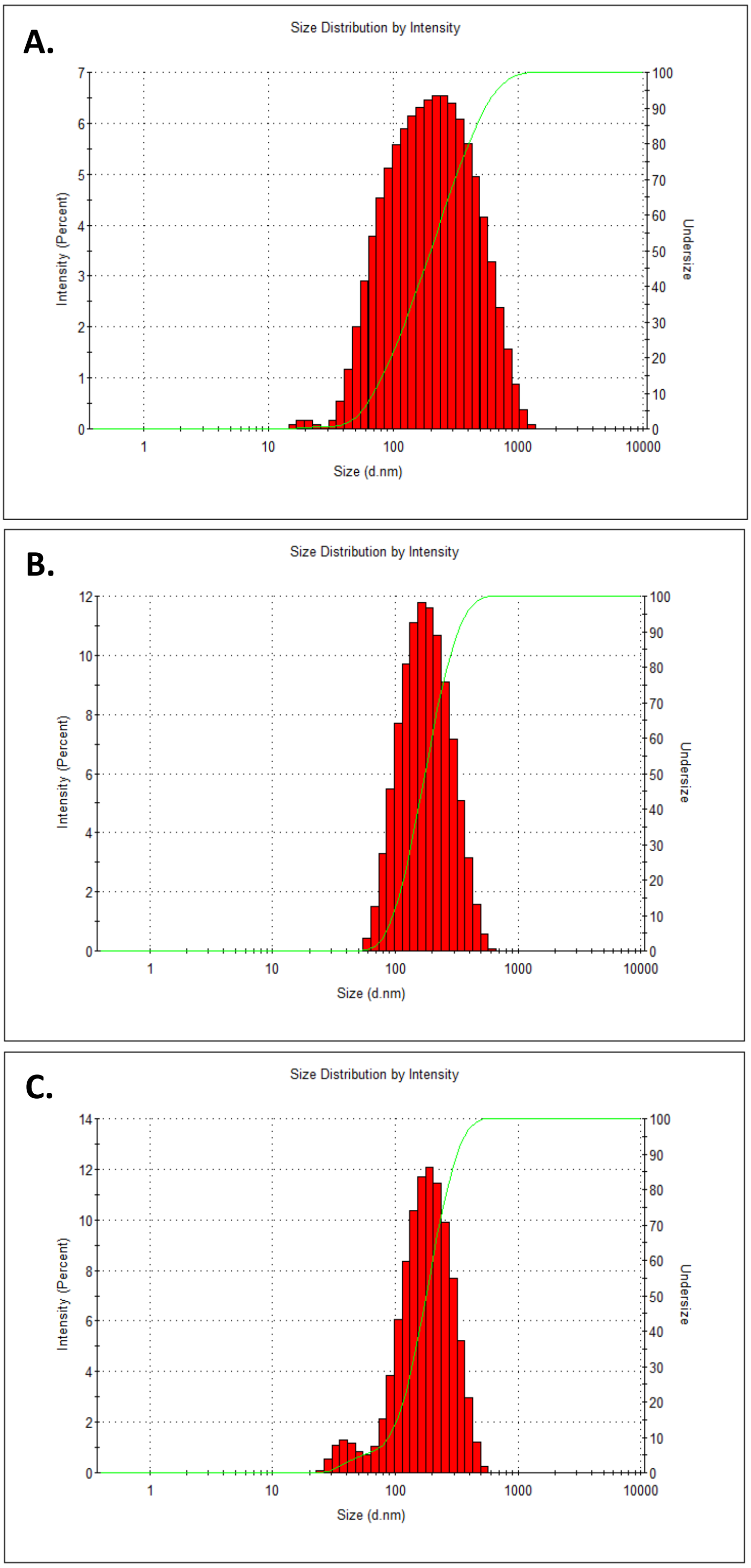

Figure S2. Size distributions from DLS, based on the scattered intensity of nanoparticles of A. EPC, B. EPC:PDMAEMA-b-PLMA 1 and C. EPC:PDMAEMA-b-PLMA 2.

Table S1. Calorimetric heating and cooling profiles of chimeric bilayers in PBS (pH = 7.4) and acidic environment (pH = 4.5).

Sample	Process	Dispersion Medium	$T_{onset,m}$ (°C)	T_m (°C)	$\Delta T_{1/2,m}$ (°C)	ΔH_m (J g^{-1})	$T_{onset,s}$ (°C)	T_s (°C)	$\Delta T_{1/2,s}$ (°C)	ΔH_s (J g^{-1})
DPPC	Heating	PBS (pH=7.4)	40.78	41.33	0.90	-53.11	34.49	36.44	1.88	7.18
DPPC:Tram-34	Heating	PBS (pH=7.4)	39.27	40.89	1.47	-56.96	-	-	-	-
DPPC:PDMAEMA-b-PLA3	Heating	PBS (pH=7.4)	40.48	41.05	0.90	-28.42	32.97	35.42	2.55	1.73
DPPC:PDMAEMA-b-PLA3:Tram-34	Heating	PBS (pH=7.4)	37.32	39.68	2.76	-34.17	-	-	-	-
DPPC:PDMAEMA-b-PLA4	Heating	PBS (pH=7.4)	40.44	41.11	0.99	-19.87	33.98	35.94	2.42	0.96
DPPC:PDMAEMA-b-PLA4:Tram-34	Heating	PBS (pH=7.4)	38.38	40.14	1.66	-24.94	-	-	-	-
DPPC	Heating	pH=4.5	40.95	41.53	1.07	-60.34	33.79	35.58	2.27	5.69
DPPC:Tram-34	Heating	pH=4.5	40.56	41.50	1.03	-39.57	-	-	-	-
DPPC:PDMAEMA-b-PLA3	Heating	pH=4.5	39.96	40.98	1.12	-37.96	-	-	-	-
DPPC:PDMAEMA-b-PLA3:Tram-34	Heating	pH=4.5	38.88	40.47	1.55	-22.71	-	-	-	-
DPPC:PDMAEMA-b-PLA4	Heating	pH=4.5	40.07	41.11	1.18	-13.14	-	-	-	-
DPPC:PDMAEMA-b-PLA4:Tram-34	Heating	pH=4.5	37.81	40.08	2.35	-21.25	-	-	-	-
DPPC	Cooling	PBS (pH=7.4)	40.11	39.57	0.97	56.81	-	-	-	-
DPPC:Tram-34	Cooling	PBS (pH=7.4)	39.84	38.75	1.83	58.69	-	-	-	-
DPPC:PDMAEMA-b-PLA3	Cooling	PBS (pH=7.4)	39.90	39.36	0.92	28.30	-	-	-	-
DPPC:PDMAEMA-b-PLA3:Tram-34	Cooling	PBS (pH=7.4)	39.29	36.97	3.65	32.83	-	-	-	-
DPPC:PDMAEMA-b-PLA4	Cooling	PBS (pH=7.4)	39.71	39.05	1.06	20.77	-	-	-	-
DPPC:PDMAEMA-b-PLA4:Tram-34	Cooling	PBS (pH=7.4)	39.36	37.76	2.27	25.66	-	-	-	-
DPPC	Cooling	pH=4.5	40.27	39.70	1.16	62.18	-	-	-	-
DPPC:Tram-34	Cooling	pH=4.5	40.48	39.64	1.38	40.65	-	-	-	-
DPPC:PDMAEMA-b-PLA3	Cooling	pH=4.5	39.86	39.18	1.34	40.09	-	-	-	-
DPPC:PDMAEMA-b-PLA3:Tram-34	Cooling	pH=4.5	39.73	38.77	1.79	22.79	-	-	-	-
DPPC:PDMAEMA-b-PLA4	Cooling	pH=4.5	39.90	38.96	1.54	17.55	-	-	-	-
DPPC:PDMAEMA-b-PLA4:Tram-34	Cooling	pH=4.5	39.48	37.49	3.00	22.56	-	-	-	-
